# Dynamics of mRNA and polysomal abundance in early 3T3-L1 adipogenesis

**DOI:** 10.1186/1471-2164-15-381

**Published:** 2014-05-18

**Authors:** Silvia von der Heyde, Carolin Fromm-Dornieden, Gabriela Salinas-Riester, Tim Beissbarth, Bernhard G Baumgartner

**Affiliations:** Department of Medical Statistics, Statistical Bioinformatics, University Medical Center Göttingen, Humboldtallee 32, 37073 Göttingen, Germany; Institute for Research in Operative Medicine (IFOM), Witten/Herdecke University, Ostmerheimer Straße 200, 51109 Cologne, Germany; Department of Developmental Biochemistry, DNA Microarray and Deep-Sequencing Facility Göttingen, University of Göttingen, Justus-von-Liebig-Weg 11, 37077 Göttingen, Germany; Department of Internal Medicine, Metabolic Diseases and Medical Molecular Biology, Paracelsus Private Medical University Salzburg, Müllner Hauptstr 48, 5020 Salzburg, Austria

**Keywords:** Adipogenesis, Transcription, Translation, 3T3-L1 pre-adipocytes, Gene ontology, Gene enrichment, Differential expression, TOP motif

## Abstract

**Background:**

Adipogenesis is a complex process, in which immature pre-adipocytes change morphology, micro-anatomy and physiology to become mature adipocytes. These store and accumulate fat and release diverse hormones. Massive changes in protein content and protein composition of the transforming cell take place within a short time-frame.

In a previous study we analyzed changes in the abundance of free and polysomal, i.e. ribosome bound, RNAs in the first hours of adipogenesis in the murine cell line 3T3-L1. Here we analyze changes of mRNA levels and their potential contribution to the changing protein pool by determination of mRNA levels and ribosome binding to mRNAs in 3T3-L1 cells stimulated for adipogenesis. We grouped mRNA species into categories with respect to up- or down-regulated transcription and translation and analyzed the groups regarding specific functionalities based on Gene Ontology (GO).

**Results:**

A shift towards up-regulation of gene expression in early adipogenesis was detected. Genes up-regulated at the transcriptional (TC:up) and translational (TL:up) level (TC:up/TL:up) are very likely involved in control and logistics of translation. Many of them are known to contain a TOP motif. In the TC:up/TL:unchanged group we detected most of the metal binding proteins and metal transporters. In the TC:unchanged/TL:up group several factors of the olfactory receptor family were identified, while in TC:unchanged/TL:down methylation and repair genes are represented. In the TC:down/TL:up group we detected many signaling factors. The TC:down/TL:unchanged group mainly consists of regulatory factors.

**Conclusions:**

Within the first hours of adipogenesis, changes in transcriptional and translational regulation take place. Notably, genes with a specific biological or molecular function tend to cluster in groups according to their transcriptional and translational regulation.

**Electronic supplementary material:**

The online version of this article (doi:10.1186/1471-2164-15-381) contains supplementary material, which is available to authorized users.

## Background

Cell transformation is a process, which requires a highly coordinated change of gene expression and subsequent structural changes of cell architecture and metabolism. First changes typically occur within very short periods of time [1], indicating that response to external stimuli initializes changes of protein activity as well as changes of protein amount via removal or *de novo* synthesis. For the regulation of protein quantity, two mechanisms are responsible: control of mRNA steady state levels by transcription and degradation, and protein synthesis by translation. There are several mechanisms known to influence transcriptional activity, the survival of mRNAs or the efficiency by which they are translated into protein [2, 3]. For translation, ribosomes attach to mature mRNAs. The more ribosomes are attached to mRNA, the more efficiently it is translated. To increase protein *de novo* synthesis, either an increase in mRNA abundance or an increase in ribosomes attached to the mRNA is required [4]. A motif typically found in the 5′-UTR of ribosomal protein (RP) mRNAs, whose translation efficiency upon stimulation depends on growth conditions, is the terminal oligopyrimidine tract (TOP) motif [5].

We applied a method to measure changes in the abundance of free mRNAs, i.e. without any ribosomes attached, and polysomal mRNAs, i.e. with one or more ribosomes attached, within the first six hours of adipogenesis in 3T3-L1 cells, a widely used model for the study of adipogenesis and function of adipocytes [6–8].

Adipogenesis is a complex process, and a number of factors are important for its regulation. Many genes are already known to play a role in the control of cell remodeling in adipogenesis [9, 10]. Despite this fact, little is known about the cellular mechanisms leading to stimulation of differentiation. Understanding these effects will be essential in order to understand the mechanism, how a mesenchymal cell decides to divide or to differentiate, which in turn will allow developing effective strategies to control fat pad growth and fight the obesity pandemic.

In a previous study we used 3T3-L1 cells mainly for the analysis of translational efficiency during the first six hours of adipogenesis [11]. Here we expand this analysis and focus on changes in total mRNA steady state levels as well as changes of the abundance of polysomal mRNAs, based on the data collected in the original experiment.

## Results and discussion

**Figure 1:**
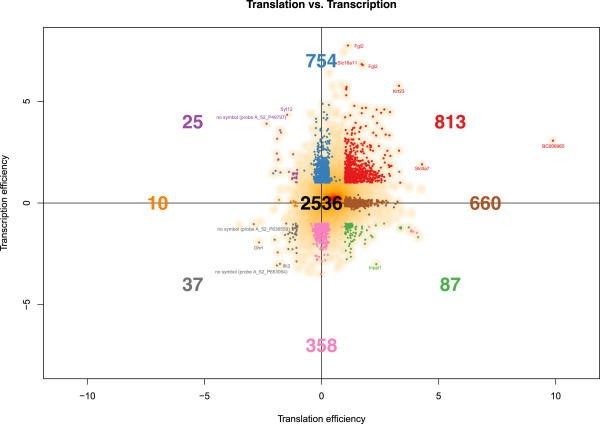
**Changes in transcriptional and translational activity.** The smooth scatter plot shows the genes of groups 1–9 in Table 1 with some selected outliers being highlighted.

Grouping genes by their translation and transcription efficiencies was based on the approach applied in [11]. As described in the methods section, we revealed differential gene expression by significant differences between time-related, namely 0 h and 6 h after hormonal stimulation, fold changes of polysomal and non-polysomal RNA fractions. We refer to the ratio of these time-related fold changes as “translation efficiency” (TL), with positive values indicating an up-regulated translation. “Transcription efficiency” (TC) is defined as the ratio of the total, i.e. polysomal and non-polysomal, fractions six hours after and before adipogenesis induction, with positive values indicating an up-regulated transcription. Differential expression was detected in an analogous manner as for translation efficiency, applying a moderated gene-by-gene t-test followed by p-value adjustment for multiple testing. We regarded differential expression as significant in case of an fdr smaller than 0.05 and an absolute log2 fold-change greater than one. Accordingly, we found nine groups of genes (Table 1), namely with significantly up-regulated transcription and translation (TC:up/TL:up), significantly up-regulated transcription and no significant translation (TC:up/TL:unchanged), significantly up-regulated transcription and down-regulated translation (TC:up/TL:down), no significant transcription and significantly up-regulated translation (TC:unchanged/TL:up), no significant transcription or translation (TC:unchanged/TL:unchanged), no significant transcription and significantly down-regulated translation (TC:unchanged/TL:down), significantly down-regulated transcription and up-regulated translation (TC:down/TL:up), significantly down-regulated transcription and no significant translation (TC:down/TL:unchanged), and significantly down-regulated transcription and translation (TC:down/TL:down). As shown in Figure 1, the groups containing most of the genes are TC:up/TL:up (813 genes) and TC:up/TL:unchanged (754). Fewer genes were found in the group TC:unchanged/TL:up (660). 2,536 genes were found to be unchanged both at the transcriptional as well as the translational level. A general shift towards increased transcriptional and translational activity has been detected (Figure 1). We detected 482 genes being significantly down-regulated at the transcriptional level (87 TC:down/TL:up, 358 TC:down/TL:unchanged, 37 TC:down/TL:down), and 1,592 genes being up-regulated at the transcriptional level (813 TC:up/TL:up, 754 TC:up/TL:unchanged, 25 TC:up/TL:down). 1,560 genes were up-regulated at the translational level (813 TC:up/TL:up, 660 TC:unchanged/TL:up, 87 TC:down/TL:up), and 72 genes were down-regulated at the translational level (25 TC:up/TL:down, 10 TC:unchanged/TL:down, 37 TC:down/TL:down).

**Table 1:** **Classification of transcripts according to their regulation at the transcriptional or translational level**

Group	Name	Transcription efficiency	Translation efficiency	#mRNAs
1	TC:up/TL:up	Up-regulated	Up-regulated	813
2	TC:up/TL:unchanged	Up-regulated	No significant change	754
3	TC:up/TL:down	Up-regulated	Down-regulated	25
4	TC:unchanged/TL:up	No significant change	Up-regulated	660
5	TC:unchanged/TL:down	No significant change	Down-regulated	10
6	TC:down/TL:up	Down-regulated	Up-regulated	87
7	TC:down/TL:unchanged	Down-regulated	No significant change	358
8	TC:down/TL:down	Down-regulated	Down-regulated	37
9	TC:unchanged/TL:unchanged	No significant change	No significant change	2,536

A total of 1,112 genes were found to be controlled only at the transcriptional level (358 down-regulated, 754 up-regulated), while 670 genes were controlled only at the translational level (660 up-regulated, 10 down-regulated).

We analyzed these groups for clusters of molecular function (MF) or biological processes (BP), using the topGO software for Gene Ontology (GO) analysis [12].

### TOP motif analysis

The 5′ terminal oligopyrimidine tract (5′TOP) motif is typical for mRNAs encoding proteins associated with the translational apparatus. This motif embeds the core of the translational cis-regulatory element of these so-called TOP mRNAs. The TOP motif is found in mRNAs coding for many components of the translational machinery. The related TOP mRNA protein synthesis is regulated in a cell growth dependent manner. To avoid energy wastage upon growth arrest or shortage of amino acids, cells repress ribosome biogenesis via translational control of TOP mRNAs by trans-acting factor(s) recognizing the 5′TOP motif (reviewed in [5]).

Hamilton et al. [13] assume that growth factors affect TOP mRNA translation via PI3K/mTOR pathways. Above that they speculate that TOP-like regulation is not restricted to mRNAs that are involved in ribosome biogenesis. Thoreen et al. [14] confirm that mRNAs, which are specifically regulated by mTORC1, consist almost entirely of transcripts with 5′TOP motifs. Furthermore they support the hypothesis that TOP and TOP-like motifs are more numerous than previously assumed.

Due to their important regulatory function in translation, TOP mRNAs may be expected to be up-regulated at least at the translational level at the first stage of adipogenesis here.

Fisher’s exact test results showed that only in the TC:up/TL:up group TOP motifs appeared significantly (p < 0.05) more often than in the other groups. The UTRscan [15] result for the related 23 genes of this group is displayed in Table 2 with two genes containing two consecutive TOP motifs each, namely Rpl30 and Polr2h. To the best of our knowledge, these data demonstrate up-regulation of TOP mRNAs in adipogenesis for the first time.

**Table 2:** **Genes bearing a TOP motif within the TC:up/TL:up group**

RefSeq	Symbol	Sequence length	TOP start and end positions inside the sequence	Matched nucleotide sequence along the sequence
NM_001042672	Prei4	205	1,6	C CTTC G
NM_010072	Dpm1	26	1,6	C TTCC G
NM_025587	Rps21	82	1,6	C TCCT G
NM_025592	Rpl35	45	1,12	C TCTTTCTCTC G
NM_027015	Rps27	33	1,8	C CTTTCC G
NM_027193	Dph5	118	1,5	C TCT G
NM_009096	Rps6	41	1,10	C TCTTTTTC G
NM_026147	Rps20	114	1,8	C CTTTCT G
NM_009419	Tpst2	168	1,5	C CTC G
NM_172086	Rpl32	51	1,11	C TTCTTCCTC G
NM_009081	Rpl28	42	1,9	C TCTTTCC G
NM_026020	Rplp2	59	1,7	C CTTTC G
NM_170669	Rps15a	17	1,9	C TTCCCTC G
NM_145596	Gatad2a	540	1,6	C CCTC G
NM_025974	Rpl14	22	1,7	C TTCTC G
NM_207625	Acsl4	460	1,8	C TTTTCC G
NM_025313	Atp5d	151	1,6	C CTTC G
NM_153529	Nrn1	164	1,8	C TTCCTC G
NM_178668	E430028B21Rik	294	1,6	C CCTC G
NM_009083	Rpl30	180	1,12	C TTCCTTTCTC G
NM_009083	Rpl30	180	13,19	C TCCCC G
NM_013481	Bop1	48	1,6	C TCCC G
NM_145632	Polr2h	96	1,5	C TCT G
NM_145632	Polr2h	96	6,10	C CCT G
NM_016844	Rps28	26	1,10	C TCCTCTCC G

### Increased mRNAs steady state levels

#### TC:up/TL:up (“translation group”)

**Figure 2:**
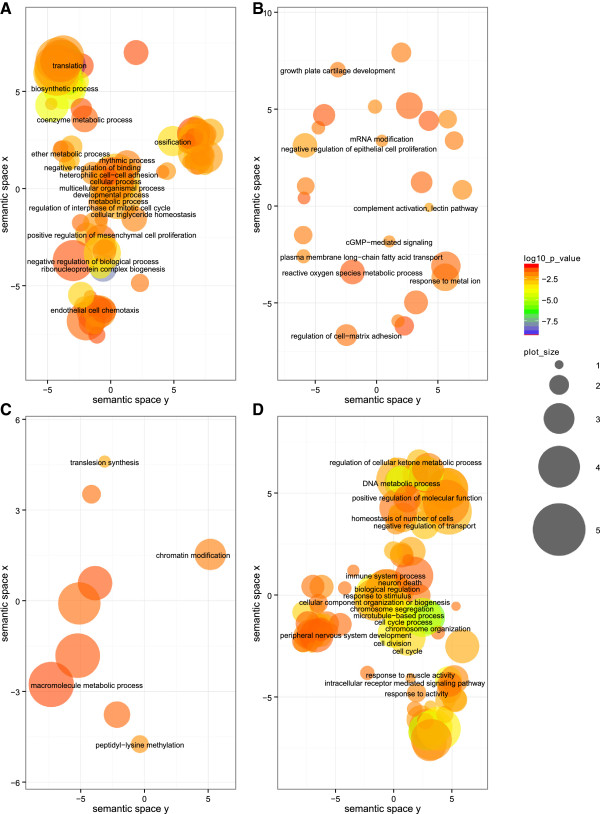
**GO term visualization (BP) for gene groups of interest.** The scatterplot shows the terms after redundancy reduction via REViGO, representing functional clusters. The bubble color indicates the p-values of the topGO analysis, and the bubble size indicates the frequency of the GO term in the underlying GOA database, i.e. the more general the term, the larger the bubbles. The GO terms cluster together in the semantic space according to functional similarity, without intrinsic meaning of semantic space units. **A**: TC:up/TL:up (“translation group”), **B**: TC:up/TL:down (“thrombospondin group”), **C**: TC:unchanged/TL:down (“methylation and repair group”), **D**: TC:down/TL:unchanged (“regulation group”).

Among the 344 inferred BP classes, the highest significant GO classes comprise ‘translation’, ‘ribosome biogenesis’, ‘rRNA processing’, ‘gene expression’, ‘macromolecule biosynthetic process’, ‘non-coding RNA (ncRNA) processing’, and ‘negative regulation of transcription from RNA polymerase II promoter’ (for the complete list see Additional file 1). The most significant groups are involved in the biogenesis of ribosomes or initiation of translation (Figure 2A). The majority of genes are involved in biosynthetic processes and regulation of cellular metabolic processes. Other groups comprise cell differentiation, cellular component biogenesis, regulation of intracellular protein kinase cascade and anatomical structure development.

Several RPs were found in about 30% of all BP classes, and IL11 was found in about 10% of all BP classes. Cebpb was detected in about 20% of all BP classes. Finally, Vegfa, Wnt5a, Sox9 and Cav1 are represented in more than one third of the 344 BP classes.

Recently, Elias et al. noted that Vascular endothelial growth factor A (VEGF-A), a classical key factor in angiogenesis and tissue remodeling, seems to play a role in the control of energy metabolism and adipose tissue function, too [16]. The Wnt pathway has also already been shown to be involved in regulation of adipocyte function [17]. Sox9 and caveolin-1 are known as well to have important functions in adipocytes or their differentiation [18, 19]. The role of Cebpb in adipogenesis has been discussed in our previous study [11]. Interleukin 11 signaling has also been regarded as regulatory functional in 3T3-L1 adipocytes [20].

Genes encoding RPs were expected to be found in the TC:up/TL:up group, as translation implies an increased abundance of ribosomes. It is speculated that all RP mRNAs belong to the class of TOP mRNAs [5].

With respect to MF, 45 GO classes were inferred, of which ‘structural constituent of ribosome’ and ‘structural molecule activity’ were found to be most significant, and the majority of genes is involved in these groups (see Additional file 2). It should be mentioned that all mRNA species in the first group (56 mRNAs) were also found in the second group (68 mRNAs). In the latter group, additionally gamma-actin, caveolin-1, cytokeratins, mitogen-activated protein kinase 8 interacting protein 3 and beta-tubulins are present. The other MF classes in the TC:up/TL:up group, as ‘endonuclease activity’, ‘polysaccharide binding’, or ‘nuclease activity’, show p-values between 0.002 and 0.04. The ‘nucleic acid binding transcription factor activity’ GO class contains 41 different mRNA species. Five genes are represented by two transcripts, amongst those Junb, Cebpb and Myc. Tsc22d3/Gilz is represented by three transcripts.

Tsc22d3/Gilz is a glucocorticoid responsive leucine zipper transcription factor, which inhibits adipogenesis in stably transfected cell lines. It binds to C/EBP binding sites in the PPARγ2 promoter and inhibits its transcription. During adipogenesis of 3T3-L1 cells, GILZ protein is detectable three hours after stimulation of adipogenesis and decreases after 24 hours, presumably allowing PPARγ2 expression at later time points [21].

Within the TC:up/TL:up group, almost all factors of the late cornified envelope (LCE) family 1 (Lce1e, Lce1f, Lce1g, Lce1h, Lce1i), that are present in the nine analyzed groups (Table 1), were found, hence changing expression in early adipogenesis. Exceptionally, Lce1m is up-regulated at the translational level but unchanged at the transcriptional level. Jackson et al. found out that LCE1 genes were predominantly expressed in human fetal, arm, penal, and abdominal skin [22]. They described an up-regulation of LCE1 genes in normal human keratinocytes by ultraviolet irradiation. Otherwise the LCE1 protein family is significantly down-regulated in aging [23]. To the best of our knowledge, a correlation between the LCE family and adipogenesis has not been described up to now.

#### TC:up/TL:unchanged (“metal group”)

103 BP classes were identified (see Additional file 3). The ‘protein modification by small protein conjugation or removal’ group, which contains all mRNAs of the ‘protein polyubiquitination’ and ‘protein ubiquitination’ groups, shows the most significant p-value. The majority of the genes are involved in transport, localization and macromolecule modification processes, which play an important role in re-organization of the cell. Other GO terms contributing to this were found, namely ‘actin cytoskeleton reorganization’, ‘Golgi vesicle transport’, ‘post-Golgi vesicle-mediated transport’, ‘Golgi to plasma membrane transport’, ‘organelle fusion’, ‘positive regulation of cell differentiation’, ‘regulation of cell-cell adhesion’ and ‘cell-cell adhesion mediated by integrin’.

Furthermore, BP classes comprise GO terms of anabolic and catabolic processes, such as ‘oxidative phosphorylation’, ‘mitochondrial electron transport, NADH to ubiquinone’ or ‘energy reserve metabolic process’. Polyamines, which are also represented, were shown to be involved in adipogenesis before [24].

**Figure 3:**
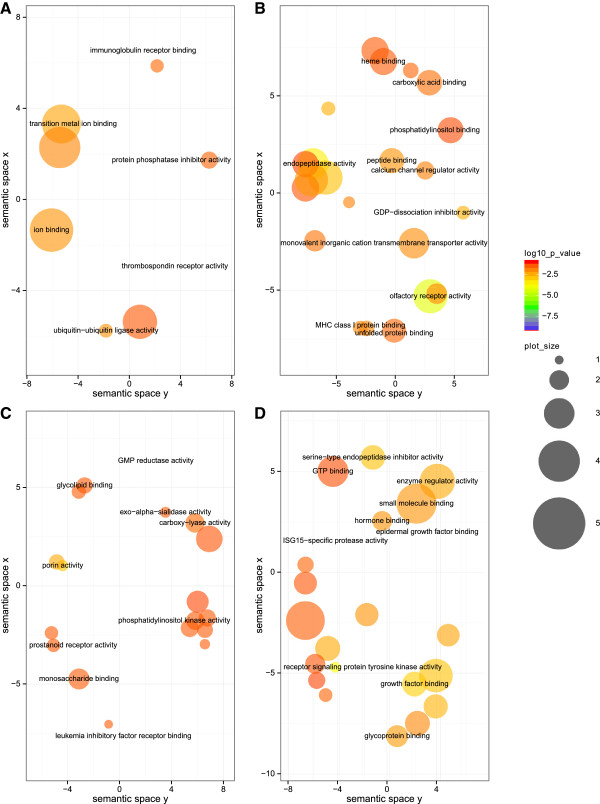
**GO term visualization (MF) for gene groups of interest.** The scatterplot shows the terms after redundancy reduction via REViGO, representing functional clusters. The bubble color indicates the p-values of the topGO analysis, and the bubble size indicates the frequency of the GO term in the underlying GOA database, i.e. the more general the term, the larger the bubbles. The GO terms cluster together in the semantic space according to functional similarity, without intrinsic meaning of semantic space units. **A**: TC:up/TL:unchanged (“metal group”), **B**: TC:unchanged/TL:up (“olfactory group”), **C**: TC:down/TL:up (“signaling group”), **D**: TC:down/TL:down (“Egfr and Ghrl group”).

The 17 MF classes are mainly related to metal binding (Figure 3A), such as ‘transition metal ion binding’, ‘ion binding’, ‘cation binding’, with a special emphasis on zinc binding (see Additional file 4). Furthermore, ‘ubiquitin-ubiquitin ligase activity’ and ‘thrombospondin receptor activity’ are represented in this group.

Zinc has been associated with adipogenesis before. Zinc levels were shown to influence the osteogenic and adipogenic differentiation of primary bone marrow stromal cells (MSC) in mouse and to inhibit adipogenesis [25]. Zinc also exerts insulin-mimetic and anti-diabetic effects. In rodent models of diabetes, Zinc treatment improves carbohydrate and lipid metabolism. In cell culture, it enhances glucose transport, glycogen and lipid synthesis, and inhibits gluconeogenesis and lipolysis [26].

An interesting factor depending on Zinc is Zinc α2-glycoprotein (ZAG). Its mRNA steady state levels in fat tissue depend on the degree of insulin resistance (IR) in morbidly obese patients [27]. In serum, ZAG correlated positively with HDL cholesterol and adiponectin and inversely with the degree of IR [28].

Zinc finger proteins (ZFP) are important regulators of transcription, and an increasing number is found to be involved in the regulation of normal cell growth and development [29]. For example, Zfp521 [30], Zfp423 [31], ZNF638 [32], ZNF395 [33], Snail [34] and kruppel-like transcription factors [35] were shown to be regulative in adipogenesis. In this group here, we detected eleven different ZFPs as well as Klf10, Klf9 and Klf5. In line with the idea that translational control plays an important role in adipogenesis, haematopoietic zinc finger (HZF) regulates the central adipogenic factor C/EBPα [36] at the translational level.

Zinc seems to play an important role in both, adipogenesis and function of mature fat tissue. Up-regulation of Zinc- and other metal-transporters was detected in our study at the transcriptional level but not at the translational level, which might indicate that their increased levels are required at later stages of adipogenesis in a regulatory manner.

#### TC:up/TL:down (“thrombospondin group”)

We identified 115 BP classes with five genes (Additional file 5, Figure 2B) and 23 MF classes with four genes (Additional file 6) being involved. Amongst those, Thbs1, Adarb1/Adar2 and Masp2 participate in both GO categories. Thrombospondin-1 (Thbs1) seems to play an important role, as it is involved in 89% of all BP groups and in 61% of all MF groups with a two-fold transcriptional up-regulation. In growth hormone induced adipogenesis of 3 T3-F442A pre-adipocytes, Thbs1 expression increased within 60 minutes, and for this it is expected to be important in initiation of adipogenic differentiation [37]. Additionally, up-regulation of thrombospondin-1 protein levels has already been shown to be associated with obesity but can be corrected by physical exercise [38]. In their recent work Kong et al. described its regulatory role in adiposity and metabolic dysfunction by enhancing adipose inflammation and stimulating adipocyte proliferation [39].

While Adarb1/Adar2 is up-regulated at the transcriptional and down-regulated at the translational level, another member of the double stranded RNA adenosine deaminases, Adarb2/Adar3, is unchanged at the transcriptional but up-regulated at the translational level. A polymorphism in Adarb2/Adar3 has recently been found to be positively associated with abdominal circumference, body mass index, serum triglyceride level, and negatively associated with serum adiponectin level [40].

### Unchanged mRNAs steady state levels

#### TC:unchanged/TL:up (“olfactory group”)

Within the 60 identified BP classes, we found several redundant but highly significant GO groups, containing different factors of the olfactory receptor family (Additional file 7). Many genes are involved in ‘G-protein coupled receptor signaling pathway’ and ‘oxidation-reduction process’. Furthermore, we detected groups of ‘vacuole organization’ and ‘vacuolar transport’, ‘glucose homeostasis’, ‘fructose metabolic process’ and ‘macroautophagy’.

Within the 47 MF classes, ‘olfactory receptor activity’ shows highest significance by far (p-value = 5.2e-05) (Additional file 8, Figure 3B). Other GO classes contain factors of ‘peptidase activity’, ‘G-protein coupled receptor activity’, ‘oxidoreductase activity’, in which most of the genes are involved, ‘acetyl-CoA C-acetyltransferase activity’, ‘glutamate receptor activity’ or ‘ion transmembrane transporter activity’. Hence, there is evidence for involvement of the olfactory system in adipogenesis.

Indeed, Åkerblad et al. [41] state that the olfactory system participates in the regulation of food intake impacting overall metabolism. They specifically analyzed the transcription factor Olf-1/early B-cell factor (O/E-1), which is important for controlling B-lymphocyte-specific genes and transcriptional regulation of genes in olfactory receptor neurons.

According to their findings, all three known O/E (Ebf) genes, which regulate genes in the olfactory system, are expressed in mouse adipose tissue and up-regulated during adipocyte differentiation. They observed that forced expression of O/E-1 in the pre-adipocyte cell line 3T3-L1 enhances adipogenesis, whereas a dominant negative form of it leads to partial suppression (reviewed in [42]).

Interestingly, O/E-1 (Ebf1) is up-regulated within less than two hours after stimulation of adipogenesis at the transcriptional level, and mRNA levels drop to normal levels approximately three hours after stimulation, followed by a slight increase of expression until 12 hours after the hormonal pulse [43]. Towards the end of adipogenesis, Ebf1 mRNA levels increase.

Here, two Ebf1 probes were spotted on the microarray. The fdr of AK036716 just slightly exceeded the cutoff of 0.05. With a translation efficiency of 0.41 and a corresponding fdr of 0.06, it was almost part of the TC:unchanged/TL:up (“olfactory”) group. The second probe (NM_007897) is part of the TC:down/TL:unchanged (“regulation”) group.

This finding might indicate that different functional populations of Ebf1 transcripts exist which are differently regulated on the transcriptional and translational level in a time-sensitive manner. The immediate decrease of Ebf1 expression after the peak at around 1 hour after hormonal stimulation explains why transcription efficiency in our analysis is not significant, as we analyzed changes between 6 and 0 hours. In the transcriptional cascade of adipogenesis, Jimenez et al. placed Ebf action between C/EBPβ/δ and C/EBPα/PPARγ, although they do not rule out further downstream effects of Ebf proteins, that might also be critical for differentiation [43].

Growth arrest at confluence is the first stage of 3T3-L1 adipocyte differentiation, and at this time also the formation of the primary cilium is induced. Zhu et al. state that the IGF-1 receptor, an essential element in inducing differentiation, is sensitized by the formation of a primary cilium in confluent 3T3-L1 pre-adipocytes [44]. Interestingly, the primary cilium is also required for G-protein-coupled olfactory receptor signaling [45], which explains the over-representation of the olfactory system in this mRNA group. If olfactory mRNAs show up-regulated translation, the primary cilium seems to be functional, and hence pinpointing to IGF-1 receptor induced differentiation of the pre-adipocytes. In this regard, it is also noteworthy that Egfr, which drives cell growth [46], was predominantly detected in the TC:down/TL:down group, thus supporting the state of growth arrest and initialization of differentiation.

The fact that G-protein coupled receptors (GPCR) mediate the detection and discrimination of diverse organic and inorganic compounds by the brain's olfactory epithelium [47], supports our finding of translationally up-regulated GPCRs here.

#### TC:unchanged/TL:down (“methylation and repair group”)

Six out of the ten genes are categorized into 18 BP classes (Additional file 9), the most frequent being MLL3, a histone methyltransferase implicated in adipogenesis [48], Rev3l, a component of zeta polymerase, which is important for translesion DNA synthesis [49] and 2210018M11Rik, according to GO analysis mainly involved in DNA repair, chromatin modification and metabolic processes. The groups ‘cellular macromolecule metabolic process’ and ‘macromolecule metabolic process’ contain all of the six genes; the group ‘nucleic acid metabolic process’ lacks only Ubr4. A graphical representation of the BP terms is shown in Figure 2C.

Five genes are distributed into 14 MF classes, with four of them being present in the group ‘nucleic acid binding’ (Additional file 10). The most significant classes include just MLL3 and are related to methyltransferase activity.

### Decreased mRNAs steady state levels

#### TC:down/TL:up (“signaling group”)

Not many genes are involved in the 191 BP classes, but almost 10% of the 87 TC:down/TL:up genes are involved in signal transduction processes, namely ‘small GTPase mediated signal transduction’, ‘regulation of protein phosphorylation’ and ‘regulation of protein serine/threonine kinase activity’ (Additional file 11). Among those genes we found Inppl1, an outlier marked in Figure 1, encoding SHIP2, which was shown to contribute to dietary obesity [50].

To get an estimate for the combined influence of changes in gene expression and protein synthesis, we calculated the sum of transcription and translation efficiency, i.e. (TC + TL)_total_. We analyzed single genes in the TC:down/TL:up group with regard to the combined influence of TC and TL. As expected, (TC + TL)_total_ was found to be positive for some factors, like Polr2a (+2.5), Pik3c2g (+2.4), Fshb (+2,2) or Zscan10 (+2.1), and negative for others, as Lbh (-1.7), Ppp1r3d (-1.4), ENSMUSG00000071724 (-1.3) or Slc40a1 (-1.2).

Vangl2 is the most abundant gene in the BP classes. It is present in 142 groups and is annotated to many pathways. In the context of adipogenesis, ‘non-canonical Wnt receptor signaling pathway’, ‘regulation of MAP kinase activity’ and ‘regulation of JUN kinase activity’ are of special interest [51, 52]. However, Vangl2 shows slight net down-regulation, i.e. (TC + TL)_total_ < 0. Other important classes are ‘cholesterol biosynthetic process’ and ‘sterol biosynthetic process’, which include the genes Mvd, Fdps and Nsdhl.

A similar situation, i.e. few genes involved in the GO classes, was observed in the 39 MF classes (Additional file 12). Here the most significant classes include ‘porin activity’ as well as important functions for glycolysis and gluconeogenesis, involving the genes Pfkm (‘6-phosphofructokinase activity’) and PCK2 (‘phosphoenolpyruvate carboxykinase activity’). Above that we detected ‘eicosanoid receptor activity’, and PPAR-γ is such a receptor [53] with important function in adipogenesis [54]. Regarding the observed signaling functions in BP, we here detected ‘phosphatidylinositol 3-kinase activity’ and ‘phosphatidylinositol phosphate kinase activity’. A graphical representation of the MF terms is shown in Figure 3C.

#### TC:down/TL:unchanged (“regulation group”)

Of the 436 BP classes, ‘chromosome organization’, ‘positive regulation of metabolic process’ and ‘positive regulation of macromolecule biosynthetic process’, ‘DNA metabolic process’, ‘organelle organization’, ‘positive regulation of transcription, DNA-dependent’, ‘microtubule cytoskeleton organization’ and ‘chromatin modification’ are among the most significant terms (Additional file 13). The majority of genes are involved in regulation, metabolism, biosynthesis and response to stimuli. A graphical representation of the BP terms is shown in Figure 2D.

64 MF classes were detected, comprising processes of lipid and nucleic acid binding, ‘RNA polymerase II transcription cofactor activity’, ‘transcription cofactor activity’, ‘chromatin binding’ or ‘RNA polymerase II regulatory region DNA binding’ (Additional file 14).

We speculate that the regulatory functions are repressed at the initialization of adipogenesis and up-regulated at later time points, which would explain the down-regulated transcription and unchanged translation of this cluster.

#### TC:down/TL:down (“Egfr and Ghrl group”)

Within the 308 BP classes, a small number of mRNAs is encountered in many groups, such as Egfr (164), Ghrl (103) or Brca2 (72) (Additional file 15). The mRNAs in this category are over-represented in ‘gland development’, ‘centrosome organization’, ‘microtubule organizing center organization’, ‘activation of MAPKK activity’, ‘response to interferon-alpha’, ‘DNA repair’ and ‘DNA replication’, ‘regulation of metabolic process’, ‘regulation of locomotion’, ‘cell cycle process’ and ‘regulation of Rap GTPase activity’. The only gene represented in ‘regulation of cyclin-dependent protein kinase activity involved in G1/S’ and ‘negative regulation of mitotic cell cycle’ is Egfr. In the group ‘G1/S transition of mitotic cell cycle’ also Pole is involved. Ghrl is, among other classes, involved in ‘regulation of appetite’, ‘sleep’, ‘response to food’ and ‘negative regulation of insulin secretion’.

The 59 MF classes comprise ‘epidermal growth factor binding’, ‘ribonucleotide binding’, ‘actin binding’, ‘protein kinase activity’ and ‘histone acetyltransferase activity’ (Additional file 16, Figure 3D). The term ‘epidermal growth factor-activated receptor activity’ is most significant, stressing an important role of Egfr here. Many publications have already focused on Egfr, a known oncogenic receptor tyrosine kinase inducing signaling cascades, in the context of adipogenesis, hence linking cancer and obesity. The activating growth factor of this receptor is the epidermal growth factor (EGF). Adachi et al. observed that EGF inhibits pre-adipocyte differentiation into mature adipocytes, but promotes adipogenesis in differentiated adipocytes in dose- and time-dependent manners in 3T3-L1 cells [55]. The dose dependence, i.e. high EGF concentrations inhibit, whereas low concentrations support pre-adipocyte differentiation, was also mentioned by Harrington et al. [56]. According to Nakano et al. [57], Egfr promotes proliferation but inhibits differentiation of adipocytes. Rogers et al. showed Egfr correlation with insulin sensitivity and PPARγ [58], stimulating adipogenesis in human pre-adipocytes. Harmon et al. showed that Egfr phosphorylates C/EBPβ in 3T3-L1 cells during the first 72 h of differentiation, also stimulating adipogenesis [59]. Also IGF1/Egfr crosstalk or transactivation was observed [60, 61], mediating the insulin-leptin-adiponectin axis in breast cancer tumorigenesis [62, 63]. Leptin also activates Egfr [64]. Above that obesity-linked SHIP2, which we detected in the “signaling group”, correlates with Egfr [65] and protects it from degradation [66]. With respect to diabetes, Miettinen et al. observed that down-regulated Egfr in pancreatic islets is linked to impaired postnatal beta-cell growth, which contributes to diabetes development [67]. Above that decreased Egfr binding was observed in diabetic rats [68], and decreased autophosphorylation of Egfr was reported in insulin-deficient diabetic rats [69]. Also glucocorticoids modulate insulin and Egfr activity in rats, depending on the animals’ dietary state [70].

The role of Ghrl, which it marked as an outlier in Figure 1, in adipogenesis has already been discussed in our previous study [11]. Interestingly, Ifit3 is an outlier in the TC:down/TL:down group, too (Figure 1), and we discussed the role of Ifit1 in our previous study [11].

## Conclusions

**Figure 4:**
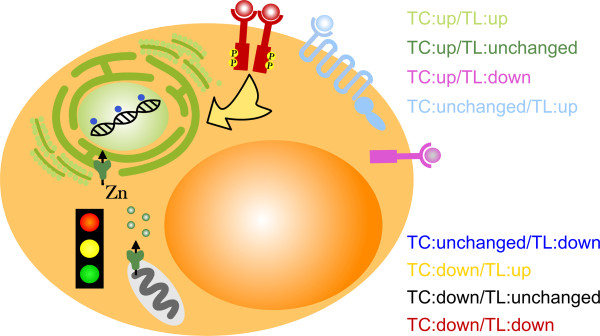
**Scheme of affected cellular processes.** The scheme of the adipocyte visualizes prominent cellular processes that are over-represented in the eight analyzed groups. The structures and name of the corresponding group have same colors. The TC:up/TL:up (“translation”) group is represented by ribosomes. The TC:up/TL:unchanged (“metal”) group is symbolized by zinc (channels). The TC:up/TL:down (“thrombospondin”) group is represented by thrombospondin binding to a receptor. Analogously, we chose receptor symbols for the TC:unchanged/TL:up (“olfactory”) group and the TC:down/TL:down (“Egfr and Ghrl”) group. The yellow arrow symbolizes signaling of the TC:down/TL:up (“signaling”) group, e.g. via phosphorylation cascades as initialized by receptor dimerization. The TC:unchanged/TL:down (“methylation and repair”) group is denoted by methyl groups attached to the DNA in the nucleus. The TC:down/TL:unchanged (“regulation”) group is represented by a traffic light.

We re-analyzed experimental data to measure changes in the transcriptional and translational activity at the beginning of adipogenesis in 3T3-L1 cells. In our previous study [11] we analyzed changes of mRNA abundance in the free mRNA and polysomal fraction after sucrose gradient centrifugation of mRNAs at time point 0 and 6 hours after hormonal induction of adipogenesis. Here, we extended the analysis of temporal changes in the abundance of polysomal mRNAs by changes in overall mRNA, i.e. transcripts, abundance and the analysis of the relation of these transcriptional and translational changes. The fact that we detected distinct functional gene clusters with specific activity status of transcription and translation at the initialization of adipogenesis points to a temporally well-orchestrated interplay between relevant genes in adipocyte transformation (Figure 4). Interestingly, main findings regarding functionalities in adipogenesis have already been published elsewhere, but here we link these biological processes and molecular functions specifically to modes of transcriptional and translational activity.

In some groups single genes are outstanding, like Egfr, linking obesity and cancer, or Thbs1, encoding the multifunctional protein Thrombospondin 1.

The majority of significant genes in the “translation group” with both, transcription and translation efficiency being up-regulated, hints at the importance of polysome organization, functionality and activity in adipocyte differentiation. In this regard, it was logically consequent to detect TOP motifs in this cluster, due to their importance in the translation regulation process.

In the context of obesity research, we intended to gain deeper insight into the transcriptional and translational interaction of genes in early adipogenesis. Based on our findings, more efficient strategies to counteract dysregulated adipocyte growth could be developed, targeting genes not only on the basis of differential expression but also on their role in specific functional clusters.

## Methods

### Gene ontology analysis

Regarding the gene groups 1–8 in Table 1, which showed at least significant transcription or translation efficiency, we were interested in enrichment for Gene Ontology (GO) terms of the classes Biological Process (BP) and Molecular Function (MF). Therefore we applied the classic gene counts based Fisher's exact test of the R package topGO [12]. Significance was defined by p-values < = 0.05. In detail, the test was performed for each of the eight gene groups with regard to the BP and MF ontology separately. This lead to the results displayed in Additional files 1, 2, 3, 4, 5, 6, 7, 8, 9, 10, 11, 12, 13, 14, 15 and 16. The eight groups contain 2,744 genes in total, of which 2,196 were annotated in the Agilent Whole Mouse Genome annotation data (chip mgug4122a, R package version 2.7.1). These represented our reference gene list. It was group-wise tested whether genes were overrepresented in a GO term, i.e. stronger represented than expected based on the reference gene list. The programming workflow and related data are provided in Additional files 17 and 18.

GO analysis results were visualized in semantic similarity-based scatterplots via REViGO [71], a web server that summarizes GO terms by removing redundant ones. The allowed similarity was chosen to be small (0.5), and the semantic similarity measure was ‘SimRel’.

### TOP motif analysis

To analyze whether 5′ terminal oligopyrimidine tract (5′ TOP) motifs occur preferentially in one of the groups of interest, namely group 1–8 in Table 1, we applied Fisher's exact test [72] for testing the null-hypothesis that the true odds ratio is not equal to one. The related contingency tables were established by counting the occurrence of TOP motifs in the respective group and the remaining ones.

To retrieve TOP motif information we used the Table Browser of the UCSC Genome Browser [73] to retrieve the sequences of the 5′ UTR exon regions for the genes (RefSeq) per group of interest, with Dec. 2011 as release date of the mouse genome assembly. Subsequently we analyzed the sequences with UTRscan [15] to detect TOP motifs.

### Cells and microarray analysis

As described in [11], we analyzed the mouse embryonic fibroblast cell line 3T3-L1 [6, 8].

In summary, 3T3-L1 pre-adipocytes were cultured to confluence and stimulated with a hormone cocktail of insulin, dexamethasone and IBMX to induce adipogenesis by standard protocols.

Cells were harvested before this hormonal induction and six hours after, when mitotic clonal expansion (MCE) and hence expression of adipogenic genes was initiated.

Polysomal mRNAs, i.e. those attached to ribosomes, were separated from free (non-polysomal) mRNAs via velocity sedimentation in sucrose gradients to analyze changes in the abundance of mRNAs in pools of both of these cell lysate fractions during the first six hours of adipogenesis.

Microarray experiments (GEO accession number GSE29744) were conducted to reveal changes in translational activity. Statistical analysis was done, using the software R [74] involving data normalization based on spike-ins and detection of differential gene expression. The latter focused on significant differences between time-related fold changes of the polysomal (p6 - p0) and non-polysomal (np6 - np0) fraction, with p0 and p6 denoting the log2 signal intensity of polysomal RNA before (p0) or six hours after (p6) hormonal stimulation, respectively. An analogous definition holds true for the non-polysomal (np) fraction.

Here we define the ratio, i.e. the difference between logarithmized data, of the mentioned time-related fold changes as “Translation efficiency” (TL = (p6 - p0) - (np6 - np0)).

In case of TL > 0, we observe an up-regulation in translation. To reveal differential expression, i.e. significant difference of TL from zero, the empirical Bayes statistic of the limma [75] R package was applied, implying a moderated gene-by-gene t-test followed by p-value adjustment via multiple testing correction according to Benjamini and Hochberg, as described in [11].

“Transcription efficiency” (TC = (p6 + np6)/2 - (p0 + np0)/2) is defined as the ratio of the total fractions six hours after and before adipogenesis induction, which means up-regulated transcription in case of TC > 0. Differential expression was detected in an analogous manner, as described for translation efficiency.

### Availability of supporting data

The data, on which our results are based on in this article, are available in the Gene Expression Omnibus (GEO) repository (accession number GSE29744).

## Authors’ information

Silvia von der Heyde and Carolin Fromm-Dornieden equally contributing first author.

Tim Beissbarth and Bernhard G. Baumgartner equally contributing senior author.

## Electronic supplementary material

Additional file 1: **BP TC:up/TL:up.** The table contains the inferred BP GO classes of the TC:up/TL:up group. (XLS 372 KB)

Additional file 2: **MF TC:up/TL:up.** The table contains the inferred MF GO classes of the TC:up/TL:up group. (XLS 25 KB)

Additional file 3: **BP TC:up/TL:unchanged.** The table contains the inferred BP GO classes of the TC:up/TL:unchanged group. (XLS 72 KB)

Additional file 4: **MF TC:up/TL:unchanged.** The table contains the inferred MF GO classes of the TC:up/TL:unchanged group. (XLS 28 KB)

Additional file 5: **BP TC:up/TL:down.** The table contains the inferred BP GO classes of the TC:up/TL:down group. (XLS 30 KB)

Additional file 6: **MF TC:up/TL:down.** The table contains the inferred MF GO classes of the TC:up/TL:down group. (XLS 10 KB)

Additional file 7: **BP TC:unchanged/TL:up.** The table contains the inferred BP GO classes of the TC:unchanged/TL:up group. (XLS 30 KB)

Additional file 8: **MF TC:unchanged/TL:up.** The table contains the inferred MF GO classes of the TC:unchanged/TL:up group. (XLS 26 KB)

Additional file 9: **BP TC:unchanged/TL:down.** The table contains the inferred BP GO classes of the TC:unchanged/TL:down group. (XLS 10 KB)

Additional file 10: **MF TC:unchanged/TL:down.** The table contains the inferred MF GO classes of the TC:unchanged/TL:down group. (XLS 9 KB)

Additional file 11: **BP TC:down/TL:up.** The table contains the inferred BP GO classes of the TC:down/TL:up group. (XLS 48 KB)

Additional file 12: **MF TC:down/TL:up.** The table contains the inferred MF GO classes of the TC:down/TL:up group. (XLS 14 KB)

Additional file 13: **BP TC:down/TL:unchanged.** The table contains the inferred BP GO classes of the TC:down/TL:unchanged group. (XLS 299 KB)

Additional file 14: **MF TC:down/TL:unchanged.** The table contains the inferred MF GO classes of the TC:down/TL:unchanged group. (XLS 36 KB)

Additional file 15: **BP TC:down/TL:down.** The table contains the inferred BP GO classes of the TC:down/TL:down group. (XLS 74 KB)

Additional file 16: **MF TC:down/TL:down.** The table contains the inferred MF GO classes of the TC:down/TL:down group. (XLS 20 KB)

Additional file 17: **Analysis dataset.** The R dataset is loaded at execution of the script provided in Additional file 18. (ZIP 5 KB)

Additional file 18: **Analysis programming script.** The R script contains the major analysis steps. It depends on Additional file 17. (ZIP 6 MB)

## Abbreviations

BP: Biological process
EGF: Epidermal growth factor
GO: Gene ontology
GPCR: G-protein coupled receptor
HZF: Haematopoietic zinc finger
IR: Insulin resistance
LCE: Late cornified envelope
MCE: Mitotic clonal expansion
MF: Molecular function
MSC: Marrow stromal cell
ncRNA: Non-coding RNA
RP: Ribosomal protein
TC: Transcription efficiency
TL: Translation efficiency
TOP: Terminal oligopyrimidine tract
ZFP: Zinc finger protein.
